# Supplementary material for: Predictors of mortality in patients with drug-resistant tuberculosis: A systematic review and meta-analysis
Source: PLoS One. 2021 Jun 28;16(6):e0253848. doi: 10.1371/journal.pone.0253848 (PMC8238236; doi:10.1371/journal.pone.0253848)
Supplement: S2 Table — (DOCX) [file pone.0253848.s002.docx]

**S2 Table: Search Engines**

| Search engine | Search string applied | Number of results for search |
| --- | --- | --- |
| Ovid Embase database | (‘predictors’/exp OR predictors OR ‘indicators’/exp OR indicators) AND (‘mortality’/exp OR mortality) AND ‘drug resistant’ AND (tuberculosis’/exp OR tuberculosis) | 56 |
| Scopus | (TITLE-ABS-KEY (predictors) OR TITLE-ABS-KEY (indicators) AND TITLE-ABS-KEY (mortality) AND TITLE-ABS-KEY (drug-resistant AND tuberculosis)) | 65 |
| Web of science | 4. # 3 AND # 2 AND #1  3. TS= (Drug-resistant tuberculosis)  2. TS= (Mortality)  1. TS= (Predictors OR indicators) | 60  8,272  984,787  1,114,856 |

**PubMed**

| Search number | Query | Sort By | Filters | Search Details | Results | Time |
| --- | --- | --- | --- | --- | --- | --- |
| 8 | ((((((associated factors) OR ("Risk Factors"[Mesh])) OR (Predictors)) OR (Indicators)) AND ("Mortality"[Mesh])) AND ("Drug Resistance"[Mesh])) AND ("Tuberculosis"[Mesh]) |  |  | (((((((((((("associate"[All Fields] OR "associated"[All Fields]) OR "associates"[All Fields]) OR "associating"[All Fields]) OR "association"[MeSH Terms]) OR "association"[All Fields]) OR "associations"[All Fields]) AND (("factor"[All Fields] OR "factor s"[All Fields]) OR "factors"[All Fields])) OR "Risk Factors"[MeSH Terms]) OR ("predictor"[All Fields] OR "predictors"[All Fields])) OR ((((((((((((("indicate"[All Fields] OR "indicated"[All Fields]) OR "indicates"[All Fields]) OR "indicating"[All Fields]) OR "indicative"[All Fields]) OR "indicatives"[All Fields]) OR "indicators and reagents"[Pharmacological Action]) OR "indicators and reagents"[MeSH Terms]) OR ("indicators"[All Fields] AND "reagents"[All Fields])) OR "indicators and reagents"[All Fields]) OR "indicator"[All Fields]) OR "indicators"[All Fields]) OR "indice"[All Fields]) OR "indices"[All Fields])) AND "Mortality"[MeSH Terms]) AND "Drug Resistance"[MeSH Terms]) AND "Tuberculosis"[MeSH Terms] | 21 | 7:35:23 |
| 7 | Indicators |  |  | (((((((((((("indicate"[All Fields] OR "indicated"[All Fields]) OR "indicates"[All Fields]) OR "indicating"[All Fields]) OR "indicative"[All Fields]) OR "indicatives"[All Fields]) OR "indicators and reagents"[Pharmacological Action]) OR "indicators and reagents"[MeSH Terms]) OR ("indicators"[All Fields] AND "reagents"[All Fields])) OR "indicators and reagents"[All Fields]) OR "indicator"[All Fields]) OR "indicators"[All Fields]) OR "indice"[All Fields]) OR "indices"[All Fields] | 3,704,732 | 7:32:15 |
| 6 | Predictors |  |  | "predictor"[All Fields] OR "predictors"[All Fields] | 384,884 | 7:31:51 |
| 5 | associated factors |  |  | (((((("associate"[All Fields] OR "associated"[All Fields]) OR "associates"[All Fields]) OR "associating"[All Fields]) OR "association"[MeSH Terms]) OR "association"[All Fields]) OR "associations"[All Fields]) AND (("factor"[All Fields] OR "factor s"[All Fields]) OR "factors"[All Fields]) | 1,619,448 | 7:31:32 |
| 4 | "Tuberculosis"[Mesh] | Most Recent |  | "Tuberculosis"[MeSH Terms] | 192,158 | 7:30:43 |
| 3 | "Drug Resistance"[Mesh] | Most Recent |  | "Drug Resistance"[MeSH Terms] | 330,882 | 7:30:12 |
| 2 | "Mortality"[Mesh] | Most Recent |  | "Mortality"[MeSH Terms] | 383,357 | 7:29:29 |
| 1 | "Risk Factors"[Mesh] | Most Recent |  | "Risk Factors"[MeSH Terms] | 828,183 | 7:28:53 |
