# Supplementary material for: Predictors of mortality in patients with drug-resistant tuberculosis: A systematic review and meta-analysis
Source: PLoS One. 2021 Jun 28;16(6):e0253848. doi: 10.1371/journal.pone.0253848 (PMC8238236; doi:10.1371/journal.pone.0253848)
Supplement: S3 Table — (DOCX) [file pone.0253848.s003.docx]

**S3 Table:** Inclusion and exclusion criteria for selection of studies to the systematic review and meta-analysis on the predictors of mortality in patients with drug-resistant tuberculosis.

| Inclusion criteria | Exclusion criteria | Description |
| --- | --- | --- |
| Original studies that specifically reported incidence of mortality and or predictors of mortality among patients with DR-TB. | Case reports | DR-TB, defined as when someone is infected with *Mycobacterium tuberculosis*, which is resistant to at least one first-line anti-TB drug |
| Studies published in English language | Studies that included a mixed population | Mixed population; when mortality rates and the predictors are used to describe mortality among DR-TB and drug susceptible TB patients within the same study /Not separately analyzed/ |
| Case control and cohort studies (both retrospective and prospective ) | Overlapped studies | Some studies are found in different versions and some studies with different publications from the same original study. |
| The studies should describe the study setting/country, the sample size, the study design, study period, study population age-group, and the number of patients who died. | Non-specific outcome | Non-specific outcome incudes studies that did not specifically reported the incidence of mortality and its predictors; i.e. some studies were reported as unfavorable/unsuccessful/poor/ treatment outcome and its predictors as a general. |
|  | Incomplete records | Incomplete records includes; letters written to editorial, proceedings, and studies with incomplete data on the number of death among the total sample size. |
