# Supplementary material for: Predictors of mortality in patients with drug-resistant tuberculosis: A systematic review and meta-analysis
Source: PLoS One. 2021 Jun 28;16(6):e0253848. doi: 10.1371/journal.pone.0253848 (PMC8238236; doi:10.1371/journal.pone.0253848)
Supplement: S4 Table — (DOCX) [file pone.0253848.s004.docx]

**S4 Table:** Quality assessment for the included studies in meta-analysis

| Author, year | Q1 | | | | Q2 | | | | | Q3 | | | | Q4 | | | | Q5 | | | | Q6 | | | | Q7 | | | | Q8 | | | | Q9 | | | | Q10 | | | | Q11 | | | | Overall quality result |
| --- | --- | --- | --- | --- | --- | --- | --- | --- | --- | --- | --- | --- | --- | --- | --- | --- | --- | --- | --- | --- | --- | --- | --- | --- | --- | --- | --- | --- | --- | --- | --- | --- | --- | --- | --- | --- | --- | --- | --- | --- | --- | --- | --- | --- | --- | --- |
|  | Y | N | U | NA | | Y | N | U | NA | Y | N | U | NA | Y | N | U | NA | Y | N | U | NA | Y | N | U | NA | Y | N | U | NA | Y | N | U | NA | Y | N | U | NA | Y | N | U | NA | Y | N | U | NA |  |
| Fantaw et al., 2018 |  |  |  | √ | |  |  |  | √ | √ |  |  |  | √ |  |  |  | √ |  |  |  | √ |  |  |  | √ |  |  |  | √ |  |  |  | √ |  |  |  | √ |  |  |  | √ |  |  |  | 9/11(81.8%) |
| Getachew et al., 2013 |  |  |  | √ | |  |  |  | √ | √ |  |  |  | √ |  |  |  | √ |  |  |  | √ |  |  |  | √ |  |  |  | √ |  |  |  | √ |  |  |  | √ |  |  |  | √ |  |  |  | 9/11(81.8%) |
| Molalign et al., 2015 |  |  |  | √ | |  |  |  | √ | √ |  |  |  | √ |  |  |  | √ |  |  |  | √ |  |  |  | √ |  |  |  | √ |  |  |  | √ |  |  |  | √ |  |  |  | √ |  |  |  | 9/11(81.8%) |
| Girum et al, 2017 |  |  |  | √ | |  |  |  | √ | √ |  |  |  | √ |  |  |  | √ |  |  |  | √ |  |  |  | √ |  |  |  | √ |  |  |  | √ |  |  |  | √ |  |  |  | √ |  |  |  | 9/11(81.8%) |
| Gebre et al., 2020 |  |  |  | √ | |  |  |  | √ | √ |  |  |  | √ |  |  |  | √ |  |  |  | √ |  |  |  | √ |  |  |  | √ |  |  |  | √ |  |  |  | √ |  |  |  | √ |  |  |  | 9/11(81.8%) |
| Woya et al., 2019 |  |  |  | √ | |  |  |  | √ | √ |  |  |  | √ |  |  |  | √ |  |  |  | √ |  |  |  | √ |  |  |  | √ |  |  |  | √ |  |  |  | √ |  |  |  | √ |  |  |  | 9/11(81.8%) |
| Balabanova et al., 2016 |  |  |  | √ | |  |  |  | √ | √ |  |  |  | √ |  |  |  | √ |  |  |  | √ |  |  |  | √ |  |  |  | √ |  |  |  | √ |  |  |  | √ |  |  |  | √ |  |  |  | 9/11(81.8%) |
| Bei et al., 2018 |  |  |  | √ | |  |  |  | √ | √ |  |  |  | √ |  |  |  | √ |  |  |  | √ |  |  |  | √ |  |  |  | √ |  |  |  | √ |  |  |  | √ |  |  |  | √ |  |  |  | 9/11(81.8%) |
| Gayoso et al., 2018 |  |  |  | √ | |  |  |  | √ | √ |  |  |  | √ |  |  |  | √ |  |  |  | √ |  |  |  | √ |  |  |  | √ |  |  |  | √ |  |  |  | √ |  |  |  | √ |  |  |  | 9/11(81.8%) |
| Kashongwe et al, 2017 |  |  |  | √ | |  |  |  | √ | √ |  |  |  | √ |  |  |  | √ |  |  |  | √ |  |  |  | √ |  |  |  | √ |  |  |  | √ |  |  |  | √ |  |  |  | √ |  |  |  | 9/11(81.8%) |
| Olaleye et al., 2016 |  |  |  | √ | |  |  |  | √ | √ |  |  |  | √ |  |  |  | √ |  |  |  | √ |  |  |  | √ |  |  |  | √ |  |  |  | √ |  |  |  | √ |  |  |  | √ |  |  |  | 9/11(81.8%) |
| Sun et al., 2015 |  |  |  | √ | |  |  |  | √ | √ |  |  |  | √ |  |  |  | √ |  |  |  | √ |  |  |  | √ |  |  |  | √ |  |  |  | √ |  |  |  | √ |  |  |  | √ |  |  |  | 9/11(81.8%) |
| Wang et al., 2019 |  |  |  | √ | |  |  |  | √ | √ |  |  |  | √ |  |  |  | √ |  |  |  | √ |  |  |  | √ |  |  |  | √ |  |  |  | √ |  |  |  | √ |  |  |  | √ |  |  |  | 9/11(81.8%) |
| Wai et al., 2017 |  |  |  | √ | |  |  |  | √ | √ |  |  |  | √ |  |  |  | √ |  |  |  | √ |  |  |  | √ |  |  |  | √ |  |  |  | √ |  |  |  | √ |  |  |  | √ |  |  |  | 9/11(81.8%) |
| Brust et al., 2018 |  |  |  | √ | |  |  |  | √ | √ |  |  |  | √ |  |  |  | √ |  |  |  | √ |  |  |  | √ |  |  |  | √ |  |  |  | √ |  |  |  | √ |  |  |  | √ |  |  |  | 9/11(81.8%) |
| Shimbre et al., 2020 |  |  |  | √ | |  |  |  | √ | √ |  |  |  | √ |  |  |  | √ |  |  |  | √ |  |  |  | √ |  |  |  | √ |  |  |  | √ |  |  |  | √ |  |  |  | √ |  |  |  | 9/11(81.8%) |
| Kanwal et al., 2017 |  |  |  | √ | |  |  |  | √ | √ |  |  |  |  | √ |  |  |  | √ |  |  | √ |  |  |  | √ |  |  |  | √ |  |  |  | √ |  |  |  | √ |  |  |  | √ |  |  |  | 7/11(63.6%) |
| Bhering et al., 2019 |  |  |  | √ | |  |  |  | √ | √ |  |  |  | √ |  |  |  | √ |  |  |  | √ |  |  |  | √ |  |  |  | √ |  |  |  | √ |  |  |  | √ |  |  |  | √ |  |  |  | 9/11(81.8%) |
| Chingonzoh et al., 2018 |  |  |  | √ | |  |  |  | √ | √ |  |  |  | √ |  |  |  | √ |  |  |  | √ |  |  |  | √ |  |  |  | √ |  |  |  | √ |  |  |  | √ |  |  |  | √ |  |  |  | 9/11(81.8%) |
| Delgado et al., 2015 |  |  |  | √ | |  |  |  | √ | √ |  |  |  | √ |  |  |  | √ |  |  |  | √ |  |  |  | √ |  |  |  | √ |  |  |  | √ |  |  |  | √ |  |  |  | √ |  |  |  | 9/11(81.8%) |
| Farley et al., 2011 |  |  |  | √ | |  |  |  | √ | √ |  |  |  |  | √ |  |  |  | √ |  |  | √ |  |  |  | √ |  |  |  | √ |  |  |  | √ |  |  |  | √ |  |  |  | √ |  |  |  | 7/11(63.6%) |
| Jeon et al., 2011 |  |  |  | √ | |  |  |  | √ | √ |  |  |  | √ |  |  |  | √ |  |  |  | √ |  |  |  | √ |  |  |  | √ |  |  |  | √ |  |  |  | √ |  |  |  | √ |  |  |  | 9/11(81.8%) |
| Kang et al., 2013 |  |  |  | √ | |  |  |  | √ | √ |  |  |  | √ |  |  |  | √ |  |  |  | √ |  |  |  | √ |  |  |  | √ |  |  |  | √ |  |  |  | √ |  |  |  | √ |  |  |  | 9/11(81.8%) |
| Kim et al., 2010 |  |  |  | √ | |  |  |  | √ | √ |  |  |  | √ |  |  |  | √ |  |  |  | √ |  |  |  | √ |  |  |  | √ |  |  |  | √ |  |  |  | √ |  |  |  | √ |  |  |  | 9/11(81.8%) |
| Kurbatovaet al., 2012 |  |  |  | √ | |  |  |  | √ | √ |  |  |  | √ |  |  |  | √ |  |  |  | √ |  |  |  | √ |  |  |  | √ |  |  |  | √ |  |  |  | √ |  |  |  | √ |  |  |  | 9/11(81.8%) |
| Makhmudova et al., 2019) |  |  |  | √ | |  |  |  | √ | √ |  |  |  | √ |  |  |  | √ |  |  |  | √ |  |  |  | √ |  |  |  | √ |  |  |  | √ |  |  |  | √ |  |  |  | √ |  |  |  | 9/11(81.8%) |
| Manda et al, 2014 |  |  |  | √ | |  |  |  | √ | √ |  |  |  | √ |  |  |  | √ |  |  |  | √ |  |  |  | √ |  |  |  | √ |  |  |  | √ |  |  |  | √ |  |  |  | √ |  |  |  | 9/11(81.8%) |
| Milanov et al., 2015 |  |  |  | √ | |  |  |  | √ | √ |  |  |  | √ |  |  |  | √ |  |  |  | √ |  |  |  | √ |  |  |  | √ |  |  |  | √ |  |  |  | √ |  |  |  | √ |  |  |  | 9/11(81.8%) |
| Mitnick et al., 2013 |  |  |  | √ | |  |  |  | √ | √ |  |  |  | √ |  |  |  | √ |  |  |  | √ |  |  |  | √ |  |  |  | √ |  |  |  | √ |  |  |  | √ |  |  |  | √ |  |  |  | 9/11(81.8%) |
| Mollel et al., 2017 |  |  |  | √ | |  |  |  | √ | √ |  |  |  | √ |  |  |  | √ |  |  |  | √ |  |  |  | √ |  |  |  | √ |  |  |  | √ |  |  |  | √ |  |  |  | √ |  |  |  | 9/11(81.8%) |
| Pradipta et al., 2019 |  |  |  | √ | |  |  |  | √ | √ |  |  |  | √ |  |  |  | √ |  |  |  | √ |  |  |  | √ |  |  |  | √ |  |  |  | √ |  |  |  | √ |  |  |  | √ |  |  |  | 9/11(81.8%) |
| Schnippel et al., 2015 |  |  |  | √ | |  |  |  | √ | √ |  |  |  | √ |  |  |  | √ |  |  |  | √ |  |  |  | √ |  |  |  | √ |  |  |  | √ |  |  |  | √ |  |  |  | √ |  |  |  | 9/11(81.8%) |
| Seifert et al., 2017 |  |  |  | √ | |  |  |  | √ | √ |  |  |  | √ |  |  |  | √ |  |  |  | √ |  |  |  | √ |  |  |  | √ |  |  |  | √ |  |  |  | √ |  |  |  | √ |  |  |  | 9/11(81.8%) |
| Seung et al., 2009 |  |  |  | √ | |  |  |  | √ | √ |  |  |  | √ |  |  |  | √ |  |  |  | √ |  |  |  | √ |  |  |  | √ |  |  |  | √ |  |  |  | √ |  |  |  | √ |  |  |  | 9/11(81.8%) |
| Suryawanshi et al., 2017 |  |  |  | √ | |  |  |  | √ | √ |  |  |  | √ |  |  |  | √ |  |  |  | √ |  |  |  | √ |  |  |  | √ |  |  |  | √ |  |  |  | √ |  |  |  | √ |  |  |  | 9/11(81.8%) |
| Dheda et al., 2010 |  |  |  | √ | |  |  |  | √ | √ |  |  |  | √ |  |  |  | √ |  |  |  | √ |  |  |  | √ |  |  |  | √ |  |  |  | √ |  |  |  | √ |  |  |  | √ |  |  |  | 9/11(81.8%) |
| Shariff et al., 2016 |  |  |  | √ | |  |  |  | √ | √ |  |  |  | √ |  |  |  | √ |  |  |  | √ |  |  |  | √ |  |  |  | √ |  |  |  | √ |  |  |  | √ |  |  |  | √ |  |  |  | 9/11(81.8%) |
| Janmeja et al., 2018 |  |  |  | √ | |  |  |  | √ | √ |  |  |  | √ |  |  |  | √ |  |  |  | √ |  |  |  | √ |  |  |  | √ |  |  |  | √ |  |  |  | √ |  |  |  | √ |  |  |  | 9/11(81.8%) |
| Samali et al, 2017 |  |  |  | √ | |  |  |  | √ | √ |  |  |  | √ |  |  |  | √ |  |  |  | √ |  |  |  | √ |  |  |  | √ |  |  |  | √ |  |  |  | √ |  |  |  | √ |  |  |  | 9/11(81.8%) |
| Rusisiro et al., 2019 |  |  |  | √ | |  |  |  | √ | √ |  |  |  | √ |  |  |  | √ |  |  |  | √ |  |  |  | √ |  |  |  | √ |  |  |  | √ |  |  |  | √ |  |  |  | √ |  |  |  | 9/11(81.8%) |
| Prajapati al.2017 |  |  |  | √ | |  |  |  | √ | √ |  |  |  | √ |  |  |  | √ |  |  |  | √ |  |  |  | √ |  |  |  | √ |  |  |  | √ |  |  |  | √ |  |  |  | √ |  |  |  | 9/11(81.8%) |
| Park et al., 2010 |  |  |  | √ | |  |  |  | √ | √ |  |  |  | √ |  |  |  | √ |  |  |  | √ |  |  |  | √ |  |  |  | √ |  |  |  | √ |  |  |  | √ |  |  |  | √ |  |  |  | 9/11(81.8%) |
| Kassa et al., 2020 |  |  |  | √ | |  |  |  | √ | √ |  |  |  | √ |  |  |  | √ |  |  |  | √ |  |  |  | √ |  |  |  | √ |  |  |  | √ |  |  |  | √ |  |  |  | √ |  |  |  | 9/11(81.8%) |
| O’Donnell et al., 2013 |  |  |  | √ | |  |  |  | √ | √ |  |  |  | √ |  |  |  | √ |  |  |  | √ |  |  |  | √ |  |  |  | √ |  |  |  | √ |  |  |  | √ |  |  |  | √ |  |  |  | 9/11(81.8%) |
| Wang et al., 2020 |  |  |  | √ | |  |  |  | √ | √ |  |  |  | √ |  |  |  | √ |  |  |  | √ |  |  |  | √ |  |  |  | √ |  |  |  | √ |  |  |  | √ |  |  |  | √ |  |  |  | 9/11(81.8%) |

****Y=yes, N=no, U=unclear, NA=not applicable, <60%=low,60-80%=medium, >80%=high quality***

Critical appraisal for case control studies

| Author, year | Q1 | | | | Q2 | | | | Q3 | | | | Q4 | | | | Q5 | | | | Q6 | | | | Q7 | | | | Q8 | | | | Q9 | | | | Q10 | | | | Overall quality result |
| --- | --- | --- | --- | --- | --- | --- | --- | --- | --- | --- | --- | --- | --- | --- | --- | --- | --- | --- | --- | --- | --- | --- | --- | --- | --- | --- | --- | --- | --- | --- | --- | --- | --- | --- | --- | --- | --- | --- | --- | --- | --- |
|  | Y | N | U | NA | Y | N | U | NA | Y | N | U | NA | Y | N | U | NA | Y | N | U | NA | Y | N | U | NA | Y | N | U | NA | Y | N | U | NA | Y | N | U | NA | Y | N | U | NA |  |
| Bajehson et al.,2019 | √ |  |  |  | √ |  |  |  | √ |  |  |  | √ |  |  |  | √ |  |  |  |  | √ |  |  |  | √ |  |  | √ |  |  |  | √ |  |  |  | √ |  |  |  | 8/10 (80%) |
| Gandhi  et al., 2012 | √ |  |  |  | √ |  |  |  | √ |  |  |  | √ |  |  |  | √ |  |  |  |  | √ |  |  |  | √ |  |  | √ |  |  |  | √ |  |  |  | √ |  |  |  | 8/10 (80%) |
| Shenoi  et al., 2012 | √ |  |  |  | √ |  |  |  | √ |  |  |  | √ |  |  |  | √ |  |  |  |  | √ |  |  |  | √ |  |  | √ |  |  |  | √ |  |  |  | √ |  |  |  | 8/10 (80%) |
| Kizito et al., 2021 | √ |  |  |  | √ |  |  |  | √ |  |  |  | √ |  |  |  | √ |  |  |  |  | √ |  |  |  | √ |  |  | √ |  |  |  | √ |  |  |  | √ |  |  |  | 8/10 (80%) |

****Y=yes, N=no, U=unclear, NA=not applicable, <60%=low,60-80%=medium, >80%=high quality***
