# Supplementary material for: Predictors of mortality in patients with drug-resistant tuberculosis: A systematic review and meta-analysis
Source: PLoS One. 2021 Jun 28;16(6):e0253848. doi: 10.1371/journal.pone.0253848 (PMC8238236; doi:10.1371/journal.pone.0253848)
Supplement: Attachment — Submitted filename: Response to Reviewers.docx [file pone.0253848.s005.docx]

**Revisions based on the Editor’s and the reviewers’ comments and suggestions**

Title: Predictors of mortality in patients with drug-resistant tuberculosis: A systematic review and meta-analysis

Editor Comments and suggestions

1. Critically, only a single manuscript version should be included in the final text.

Thank you for the valuable comment. The problem occurred due to the second manuscript submitted after the technical check comments without removing the first submission. The current revised manuscript is a single version.

2. Please take extra care to address all issues relating to the methodology

Thank you for the comment. We revised the manuscript as per the comments and suggestions that were given by the editor and the reviewers.

3. Additionally, thorough proofreading of the text is necessary

Thank you for the constructive comment. The current manuscript is revised, and proofread by someone with better English language skills.

Additional Editor Comments:

1. The mortality was associated with “older age” separately from “1 year increase in age” suggesting “older age” is a category. However “older age” is not defined?

Thank you for the valuable comment. We operationalized “older age” in the revised manuscript as; the highest age category in the individual studies and most of the studies, it is above 60 years.

2. Table 2 lists “for one year increase” at the last row of the table. While this implies age in the context of the rest of the text, it should be revised so that the table is understandable on its own.

Thank you for the comment, now revised accordingly as a one-year increase in age.

3. Some sorting of the Table 1 /Figure 2 would make the data more easy to read. For example if the studies were listed in alphabetical order, date of publication or something similar allowing easier cross referencing between table and figure.

Thank you for the comment and suggestion. In the revised manuscript, the studies in Table 1 are listed in alphabetical order.

4. While not an expert on meta analysis, it is strange that Figure 2 has a note mentioning weights, while no weighting information is shown. Figure 3 has the same note but also the “% weight“ column.

Thank you for the valuable comment. In the revised submission, all figures have weighting information

5. In the combined PDF document there are 2 versions of the manuscript, one version from page 5 to page 23. And another version from page 24 to page 39 (?). Some figures are repeated (Fig 1 at page 40 and 45. More importantly there are 2 different figures labelled Figure 4 (one at page 44 and one at page 48). One version of the manuscript specifically mentions figures 1-4 but doesn’t mention figures 5-7. Which manuscript is final?
   Figure 4= Figure 7 subpanels should be made more uniform
   Figure 6 is difficult to read.

Thank you for the valuable comment; this was due to the mix-up of two uploaded manuscripts. The current manuscript is revised accordingly. Since figure 6 is not readable due to a large number of studies, we removed it from the revised manuscript. However, we captured information. The number of figures in the revised manuscript is revised due to the suggestion of Reviewer 1 to perform a separate analysis based on the drug-resistance pattern.

6. Typos and trivial

Thank you for the valuable comment. The entire manuscript is checked for typos and proofread by someone with better English language skills.

7. The manuscript should be thoroughly proofread since it contains many typographical spacing errors starting at Line 4 with authors names

Thank you for the comment. It is corrected.

8. The word inconvenience(s) as used at lines 94, 113 should probably be replaced by inconsistencies

Thank you for the suggestion. We replaced it.

9. Line 441 – 442 font sizes are different.

Thank you for the comment. We corrected it.

Reviewer 1

Reviewer #1: In this systematic review, authors aimed to determine the predictors of mortality in patients with drug-resistant tuberculosis.

1. The major limitation of this analysis is that it does not distinguish between different types of drug resistance. They analyzed all patients with „resistance to any TB drug“ which means that patients with i.e. isoniazid- or ethambutol or PZA- monoresistance are mixed with patients with MDR and even XDR TB. Given that these are very different populations of patients with very different outcomes (according to all knowledge from the literature), it does not make sense to mix them together in order to try to have some conclusions that consequentially cannot be applied for neither on of those subgroups among the hat of „drug resistance“. Accordingly, the calculated proportion of death is 25.35, but ranges from 6.74% to 79.86%, which is, most probably, a reflection of very different populations in studied cohorts. I would suggest to differentiate patients populations and focus on a subgroup (i.e. monoresistance, MDRTB or XDR TB) in order to have information that could be useful or applied in real life.

Thank you for your valuable comment and suggestion. In the revised manuscript, we analyzed the pooled estimate of mortality for MDR-TB and XDR-TB separately.

2. Other remarks include many typo errors and a general need to improve the language of the manuscript as well as duplication of the figures in the uploaded file.

Thank you for the pertinent comment. The current version is checked by someone with better English language skills, and the duplicate figures are removed. The duplicates were due to uploading the revised manuscript after a technical check without removing the first manuscript.

Reviewer 2

Reviewer #2: Dear authors - I appreciate the opportunity to read your manuscript. I was asked to review the methods, so I will restrict my comments to that section. I have a few comments.

1. The databases searched are suitable. Please include the platforms (e.g. Ovid Embase or Elsevier Embase, etc)

Thank you for the valuable comment. We included the Ovid Embase platform in the revised manuscript.

The search string applied for the Ovid Embase database was (‘predictors’/exp OR predictors OR ‘indicators’/exp OR indicators) AND (‘mortality’/exp OR mortality) AND ‘drug resistant’ AND (tuberculosis’/exp OR tuberculosis).

2. I appreciate the attempt to search grey literature.
3. Thank you for reporting the search date. It's from June 2020. Consider an update to make the search more current.

Thank you for the valuable comment. We tried to find newly published articles but we could not find any relevant articles for the current study.

4. I appreciate the inclusion of an additional file with the searches. Rather than include screenshots of the database, please include just the searches in the document. The search terms in PubMed are cut off, for example.

Thank you for the comment. We uploaded the full search text of the PubMed search and included it in the additional file 2.

5. Each search would benefit with explicit use of keywords and controlled terms. For example, the common structure would be something like this:

#1 'drug resistant tuberculosis'/exp OR 'drug resistant tuberculosis':ti,ab
#2 'mortality'/exp OR mortalit*:ti,ab OR death*:ti,ab
#3 predict*:ti,ab OR indicat*:ti,ab
#4 #1 AND #2 AND #3

Thank you for the valuable comment. We included the keywords and controlled terms in the searching strategy part of the methods section.

6. This is for Elsevier Embase. Each one of these search lines could be expanded further with synonyms and other variations. But this brings in several times the results of the current search reported, and I don't think they'd all be too far afield. So, I recommend broadening the searches and reporting them more accessibly in the 'additional file'.

Thank you for the valuable comment. The search string we used for the Elsevier Embase was (Predictors OR indicators AND mortality AND drug-resistant AND tuberculosis). We tried to broaden the searches based on your recommendation, but we could not get additional relevant articles for the current study.

Reviewer 3

Reviewer #3: The reviewer congratulates the authors on this submission. Below are some comments for consideration.

1. Existing literature:

There are a number of previous systematic reviews looking at outcomes of drug-resistant TB, one even written by the same team as the current review (Alemu et al 2020, Poor treatment outcome and its predictors among drug-resistant tuberculosis patients in Ethiopia: A systematic review and meta-analysis.) It would be important to emphasize the value added by the new work - what is different about the current review? (If it is just that the other review focused on treatment outcomes generally, and this one is focusing on mortality, then this should be stated).

Thank you for the valuable comment. We included the justification in the current manuscript as per the suggestion.

2. Introduction:
   - Introduction is somewhat brief, could expand upon specific prior work and findings regarding risk factors for mortality among DR TB patients, and why previous systematic reviews have been insufficient / how your review adds value.

Thank you for the comment. We revised it as follows. Even though there are previously conducted systematic reviews regarding the poor treatment outcome of DR-TB and its predictors, most of the studies are geographically restricted. However, there is limited information that specifically addressed the predictors of mortality among DR-TB patients at the global level.

3. Methods:
   - Specific table of inclusion/exclusion criteria would be helpful (e.g. age range, locations of studies, etc)

Thank you for the comment and suggestion. The details of the individual studies (study country, setting, period, age-group, study design, etc) included in the study are described in Table 1. Figure 1 addressed the inclusion and exclusion criteria. In this study, there is no place and time restriction with the limitation of including only those studies published in English

- Clarify PICOS elements, e.g. for DR TB, state diagnostic criteria that participants need to fulfill in order to be included (case definition is mentioned in discussion, but should be moved up to be mentioned earlier, in methods). For outcome (mortality), state whether this was defined as death from any cause during the treatment period, or whether it was DR TB specific mortality.

Thank you for the pertinent comment. We revised it and the diagnostic criteria we used is included. “Drug-resistant tuberculosis, defined when someone is infected with *Mycobacterium tuberculosis*, which is resistant to at least one first-line anti-TB drug…already found in the methods part”. “The laboratory diagnostic methods to rule out DR-TB could be conventional phenotypic drug-susceptibility test or molecular methods like Xpert MTB/RIF assay and Line Probe Assay (MTBDR*plus*, MTBDR*sl*)”.

- Justification of using both fixed and random effects MA? Why not use random effects for all analyses given that the studies are done in different populations?

Thank you for the valid comment. We planned to perform a fixed or random effect model based on the heterogeneity, unfortunately, since the studies are from different populations all the pooled estimates were based on the random effect model.

4. Results:
   - Quality assessment results not described in results section, need to add this. In addition to description, would be good to add a visual representation, e.g. summarizing the information in Supplementary file 3.

Thank you for the comment. We revised as per the suggestion. Based on the results found through the JBI quality assessment tool, the indicators were turned in to 100% and graded as high if >80%, medium between 60–80%, and low <60%. Accordingly, the majority of the studies (44 out of 46) graded to have high quality, and only two studies were categorized under medium quality.

5. Minor comments:

- line 133: start by mentioning total number of records returned.
Thank you, we revised it.

“From the whole search, we assessed 640 articles for eligibility. After 203 studies were removed by duplication, 437 articles were screened by title and abstract. Then, 351 articles were excluded and full-text screening was conducted on 86. Accordingly, 41 studies were excluded from the study due to mixed study groups (12), overlapped studies (10), did not have the specific outcome (9) and incomplete records (9)”.

6. Discussion:
   - Need to add discussion of study quality and how this may have affected results.

Thank you for the valuable comment; we included it in the revised manuscript.

7. Tables and Figues:

- Table 1: Not sure if this is ordered in any way but if not, could order by year, country, or author's last name.

Thank you for the valuable comment and suggestion, now it is alphabetically ordered using the Authors name.

- Figure 1: Recommend to use original PRISMA version of screening flowchart. For studies excluded at full text review, reasons for exclusion need to be stated (e.g. case reports, n=X, etc). (It seems this has now been included in revised version).

Thank you for the comment. We already corrected it during the technical check.

- Figure 6 - not possible to see study author names/years, need higher quality image.

Thank you for the comment. Due to a large number of studies, it is not visible, thus we removed it from the revised manuscript. However, we capture the information.

- Figure 7 estimates difficult to see, suggest larger/higher quality images.

Thank you for the comment and suggestion. It is due to the small size figures, but its quality is good. If this manuscript is accepted the size would be decided during production.

- Additional file 2: please display specific search terms in a table rather than as a screenshot of databases. The search terms are sometimes cut-off in the screenshots, so not clear to see.

Thank you for the valuable comment; we revised it.

General comments:

- It is mentioned that all data is contained in the manuscript and supplementary files - will code be shared as well?

Thank you for the question, yes it is possible.

- in line 31, replace "electronic five major" with "five major electronic"

Thank you; now corrected.

- other minor grammar errors throughout, please verify.
- line 113: inconvenience? unclear. Maybe meant to say inconsistencies / discrepancies between reviewers?

Thank you for the question. Now corrected to inconsistencies.
