# Supplementary material for: Predictors of mortality in patients with drug-resistant tuberculosis: A systematic review and meta-analysis
Source: PLoS One. 2021 Jun 28;16(6):e0253848. doi: 10.1371/journal.pone.0253848 (PMC8238236; doi:10.1371/journal.pone.0253848)
Supplement: Attachment — Submitted filename: Response to reviewers.docx [file pone.0253848.s006.docx]

**Revisions based on the Editor’s and the reviewers’ comments and suggestions**

**Title: Predictors of mortality in patients with drug-resistant tuberculosis: A systematic review and meta-analysis**

**Editor Comments and suggestions**

1. The critical concern of reviewer 1 remains unmet after revision. Thus another reviewer was invited to additionally assess the study and raised similar concerns. The data was inappropriately combined and thus the conclusions drawn fail the publication criteria 4. <https://journals.plos.org/plosone/s/criteria-for-publication>

Therefore, we invite you to submit a revised version of the manuscript that addresses the points raised during the review process and critically address the issue of MDR and XDR data in your predictors of mortality analysis.

**Thank you for the valuable comments and suggestions. We performed a sub-group analysis on the predictors of mortality per the types of resistance categories based on the data presented in the primary studies. Besides, we broadened our searching and four primary studies included in the revised manuscript. We performed the pooled analysis by considering these new studies.**

**Reviewers’ comments and suggestions**

**Reviewer #1**

1. **Reviewer #1:** Authors have partly addressed major remarks and have done the analyse of the pooled estimate of mortality for MDR-TB and XDR-TB separately. Still, the risk factors were assessed for all DR-TB as a group. Given that these are very different populations of patients with very different outcomes (according to all knowledge from the literature), it does not make sense to mix them together as the conclusions that consequentially cannot be applied for neither on of those subgroups among the hat of „drug resistance“.

Given the amount of quality work and analysis that was put into this manuscript, it would be of a great value to do the risk analyse separately for different groups of resistance (or at **least mono-resistance** vs **MDR and XDR**) as it would have much more sense and value for every day clinical work. I would suggest at least to put a subgroup analysis in a supplemental (if the numbers of subgroups under-power the analysis)

**Thank you for the constructive comment. As per the suggestion given, we performed a sub-group analysis per the resistance category presented in the primary studies.**

**Reviewer #3:**

**Reviewer #3:** The reviewer thanks the authors for their efforts to address reviewer suggestions. Some minor comments remain unaddressed, see below:

1. Introduction:

Introduction still somewhat brief - need to discuss findings of prior reviews. Nice to see you have now mentioned that other reviews exist on the topic, but you have not cited them. Suggest to cite them where you first mention them (Line 77 of the introduction), and briefly mention their findings and what remains to be explored.

**Thank you for the comment once again. In the revised we cited some previous studies and also we presented the findings of a systematic review and meta-analysis study performed by our team in 2020 that showed the incidence of poor treatment outcome and its predictors among DR-TB patients in Ethiopia. The study also estimated the mortality incidence density rate.**

2. Methods:

As mentioned in the previous round of reviews, a table clearly listing the inclusion and exclusion criteria would be helpful. Although you have mentioned reasons for exclusion in the Figure 1 flowchart, these are vague (e.g. “mixed study groups”, “overlapped studies”). It would be good to have a table of minimum requirements for inclusion, i.e. which study designs included, minimum requirements for data reported to be included, etc…

**Thank you for the comment and suggestion. In the revised manuscript, we prepared a table for inclusion and exclusion criteria as a supplementary table 1.**

3. Additional file 2:

Please display specific search terms in a table rather than as a screenshot of databases. The author response sheet says this has been corrected but I am not sure where, as the screenshots are still in additional file 2.

**Thank you for the valuable comment. Previously we presented the PubMed search terms in the table, but in the current version, we displayed the search terms for the remaining search engines in table.**

**Reviewer #4:**

**Reviewer #4:** I have read with interest the article by Alemu and co-authors. This systematic review and meta-analysis, aiming to identify predictors of mortality for patients with multidrug-resistant tuberculosis, has already been reviewed in detail by three Reviewers. I will therefore limit my comments to high-level overall considerations.

1. In agreement with Reviewer 1, I think that the main criticism to this study is related to the inclusion of all types of drug-resistance. Pooling resistance “to at least one first-line anti-TB drug” does not make sense from a clinical perspective. I appreciate that the authors tried, in the revised version of the manuscript, to analyse separately mortality rates for MDR-TB and XDR-TB. However, this is not applied to the main objective of the study, the identification of predictors of mortality. And, this does not solve the issue of including patients with monoresistance to a TB drug (like isoniazid-monoresistance for instance) which have different mortality risk and may have different mortality predictors. My advice, as suggested by Reviewer 1, would be to restrict the systematic review to specific groups of drug resistance (for example, a) isoniazid-resistant, rifampicin-susceptible; b) rifampicin-resistant, fluoroquinolone-susceptible; and c) rifampicin- and fluoroquinolone-resistant) and identify mortality rates and predictors for ach of this groups. This way, the results would be clear and more meaningful.

**Thank you for the valuable comment and suggestion. As per the suggestion, we performed a sub-group analysis to assess the predictors of mortality among different resistance categories as presented in the primary studies.**

2. Performing and analysis stratified by clinically-relevant subgroups of drug-resistant TB patients, as suggested in point 1, would also increase the novelty and interest of the results of this study, compared to existing literature (as per comment of Reviewer 3).

**Thank you for the comment. We performed analysis as per the suggestion.**

3. The search should be updated to identify more recent studies, as noted by Reviewer 2.

**Thank you for the comment. We updated the search and we identified additional four studies. We analyzed the mortality rates and the predictors of mortality by including the additional identified studies.**
