# Supplementary material for: Predictors of mortality in patients with drug-resistant tuberculosis: A systematic review and meta-analysis
Source: PLoS One. 2021 Jun 28;16(6):e0253848. doi: 10.1371/journal.pone.0253848 (PMC8238236; doi:10.1371/journal.pone.0253848)
Supplement: Attachment — Submitted filename: Response to reviewers.docx [file pone.0253848.s007.docx]

**Revisions based on the Editor’s and the reviewers’ comments and suggestions**

**Title: Predictors of mortality in patients with drug-resistant tuberculosis: A systematic review and meta-analysis**

Editor

All reviewers commend the addition of the subgroup analysis, but 2 reviewers note that this addition was not adequately included in the results and discussion and removed from the limitations. Please try to revise at least the discussion text accordingly and mention this in the abstract at least briefly.

Reviewers' comments:

Reviewer #1: Authors have added the subgroup analyses. Few sentences regarding that should also be added in the discussion (i.e. to discuss the differences (or absence of) between the subgroups regarding the mortality predictors. Also, as this is no more a limitation of the study, the sentence under the line 281 (about not performing sub-group analysis) should be removed.

Reviewer #3: The reviewer thanks the authors for their efforts to address the reviewer’s suggestions. The reviewer has no further suggestions, as the previous suggestions have now been addressed. The reviewer recommends acceptance of the manuscript, provided remaining comments from the other reviewers have also been adequately addressed.

Reviewer #4: I thank the authors for submitting this revised version of the manuscript.
The authors should be commended for including a sub-group analysis stratified by drug resistance pattern (as requested) and for updating their search and increasing the number of papers included in the meta-analysis.

However, the sub-group analysis is currently only presented as a secondary paragraphin the results, not commented in the discussion, and not included at all in the abstract. In my opinion, what is currently presented as sub-group analysis should actually represent the main result of the study: as such, the results of the stratified analysis should be presented in the abstract and developed in the discussion section.

**Response**

Thank you to all the reviewers and the editor for the valuable comments and suggestions that improved the quality of the manuscript. At this stage, we included the predictors of mortality across different drug-resistance categories at the abstract section (line 45-47) and at the discussion section (line 276-298). We removed the sentence “Also, subgroup analysis based on the type of drug-resistance pattern was not performed.” as this is no more a limitation of the study.
